# Cyclophilin A enhances macrophage differentiation and lipid uptake in high glucose conditions: a cellular mechanism for accelerated macro vascular disease in diabetes mellitus

**DOI:** 10.1186/s12933-016-0467-5

**Published:** 2016-11-03

**Authors:** Surya Ramachandran, Anandan Vinitha, Cheranellore Chandrasekharan Kartha

**Affiliations:** Cardiovascular Diseases and Diabetes Biology, Rajiv Gandhi Centre for Biotechnology, Trivandrum, India

**Keywords:** Atherosclerosis, Macrovascular complications, Diabetes mellitus, Macrophages, Cyclophilin A, Monocyte adhesion, Transmigration, Foam cell formation, Hyperglycemia, THP 1

## Abstract

**Background:**

Vascular disease in diabetes is initiated by monocyte adhesion to vascular endothelium, transmigration and formation of foam cells. Increasing clinical evidence supports a role for the secretory protein, cyclophilin A in diabetic vascular disease. The means by which cyclophilin A contributes to vascular lesion development in diabetes is however largely unknown.

**Methods:**

In this study we investigated using THP1 cells and human monocytes whether cyclophilin A under hyperglycemic conditions, functions in the inflammatory cascade as a chemoattractant and increases lipid uptake by formation of foam cells invitro. We developed an invitro model of monocytes cultured in 20 mm glucose (high glucose) equivalent to 360 mg/dL of plasma glucose levels. These monocytes were then differentiated into macrophages using PMA and subsequently transformed to lipid laden foam cells using oxidized low density lipoproteins in the presence and absence of cyclophilin A. This cellular model was used to study monocyte to macrophage differentiation, transmigration and foam cell formation. A similar cellular model using siRNA mediated transient elimination of the cyclophilin A gene as well as chemical inhibitors were used to further confirm the role of cyclophilin A in the differentiation and foam cell formation process.

**Results:**

Cyclophilin A effectively increased migration of high glucose treated monocytes to the endothelial cell monolayer (p < 0.0001). In the presence of cyclophilin A, differentiated macrophages, when treated with oxLDL had a 36 percent increase in intracellular lipid accumulation (p = 0.01) when compared to cells treated with oxLDL alone. An increased flux of reactive oxygen species was also observed (p = 0.01). Inflammatory cytokines such as TNF-α, MCP-1 and cyclophilin A were significantly increased. Silencing cyclophilin A in THP-1 cells and human monocytes using siRNA or chemical inhibitor, TMN355 resulted in decrease in lipid uptake by 65–75% even after exposure to oxidized LDL. The expression of scavenger receptors expressed during differentiation process, CD36 and LOX-1 were decreased (p < 0.0001). Levels of extracellular cyclophilin A and other inflammatory cytokines such as TNF-α and MCP-1also significantly reduced.

**Conclusions:**

Taken together, we describe here a possible cellular basis by which cyclophilin A may accelerate atherogenesis in diabetes mellitus.

**Electronic supplementary material:**

The online version of this article (doi:10.1186/s12933-016-0467-5) contains supplementary material, which is available to authorized users.

## Background

The risk of vascular complications in patients with diabetes mellitus is altered by chronic hyperglycemia, increased reactive oxygen species and abnormal activation of several molecules. Chronic inflammatory processes in the vascular wall begins with recruitment of monocytes, increased monocyte transmigration, vascular permeability, differentiation into tissue macrophages eventually leading to formation of lipid laden foam cells. Monocytes function as sentinel cells during atherogenesis. Monocytes adhere to endothelial cells and later migrate into the subendothelial space in response to chemotactic activation. These monocytes differentiate into macrophages and take up oxidized lipids. The cells transform into cholesterol laden foam cells. These foam cells can become apoptotic and together with cellular debris contribute to fatty lesions leading to atherosclerotic plaque formation. High blood glucose levels further facilitate monocyte adhesion to endothelial cells, differentiation of monocyte into macrophages ultimately promoting atherogenesis [1], thus increasing the risk of vascular disease in diabetes mellitus. The regulatory signals for monocyte transmigration, differentiation and foam cell formation in the vascular wall in a diabetic milieu are not completely understood. In an earlier study we found that high glucose activates monocytes to secrete proteins which may increase the risk for vascular lesion formation [2]. One such protein is cyclophilin A, an immunophilin which has also been discovered to be elevated in the blood of patients with type 2 diabetes as well as patients with coronary artery disease [3, 4].

Cyclophilin A is present in monocytes [3], endothelial cells [5] and vascular smooth muscle cells [6]. It is also secreted from these cells in response to inflammatory stimuli such as hyperglycemia, hypoxia, infection and oxidative stress [5, 6]. Plasma levels of extracellular cyclophilin A correlates with anatomical severity of stable coronary artery disease [7]. Serum cyclophilin A concentration is significantly higher in subjects with unstable angina and acute myocardial infarction than in patients with stable angina and controls [8]. Cyclophilin A increases endothelial cell activation and inflammation in vascular wall cells [9]. Secreted cyclophilin A activates endothelial cells, which in turn contributes to recruitment of circulatory monocytes [10]. Overall, there is evidence that cyclophilin A can be implicated in the pathogenesis of vascular inflammation.

The purpose of this study was to establish in an invitro cellular model, the ability of cyclophilin A to induce monocyte adhesion to endothelial cells, migration of monocytes as well as formation of foam cells, to explore cellular mechanisms for accelerated atherosclerosis in diabetes mellitus. Our findings indicate a pivotal role for cyclophilin A in increasing monocyte migration and foam cell formation in the vessel wall under hyperglycemic conditions.

## Experimental procedures

### Reagents and antibodies

Phorbol 12-myristate 13-acetate (P-8139), LPS (L-5293), oil red O stain (O-1391), Hoechst 33258 (#861405), mission siRNA (EHU-107101), Histopaque (1077), Trichloro Acetic Acid (TCA) (T6399), 2 Thiobarbituric Acid (T5500), Malondialdehyde tetrabutylammonium salt (36357), Butylated hydroxytoluene (W218405) were procured from Sigma Aldrich, USA. Cyclophilin A recombinant protein (3589-CA) and TMN 355 (#4152) were purchased from R & D systems Inc., USA. Dil labeled oxidized LDL (J-64164) and dil labeled acetylated LDL (J-65597) were obtained from Alfa Aesar, USA. Mouse anti-cyclophilin A (ab-58144), anti mouse IgG HRP (ab-6789), Anti-LOX 1 antibody (ab60178), anti rabbit IgG-HRP (ab-97051) and catalase assay kit (ab83464) were purchased from Abcam, UK. Mouse anti β Actin (sc-47778) and rabbit anti-CD 36 (#14347) was purchased from Cell Signaling Technology, USA and Santa Cruz, USA respectively. Protease inhibitor cocktail tablets (#04 693 159001) was purchased from Roche, Switzerland. CD 14 MicroBeads (human) (130-050-201), CD 14—FITC, Human (clone: TUK4) (130-098-063) were purchased from Miltenyi Biotech, Germany. CellROX Deep Red Reagent (c10422) was purchased from Thermo Fisher Scientific, USA. All other reagents used for assays were of analytical grade.

### Culturing and treatment of THP cell lines and human monocytes

Monocyte cell lines, THP1 were obtained from American Type Culture Collection (ATCC No: THP1 (ATCC^®^ TIB-202™) USA. Cells were cultured in 10% RPMI 1640 medium (Gibco, USA). We developed an invitro model of monocytes cultured in 20 mM glucose equivalent to 360 mg/dL of plasma glucose levels in human subjects. Cells treated with mannitol (9.5 mM) was used as hyperosmolar control as described previously [2]. THP-1 cells were differentiated into macrophages by treating with 50 ng/mL PMA for 96 h. EA.hy926 (ATCC^®^ CRL-2922) cells were cultured in Dulbecco’s Modified Eagle’s Medium (Gibco, USA).

Primary monocytes were isolated from blood of healthy human volunteers as described earlier [2]. This study was approved by the Human Ethics Committee of Rajiv Gandhi Centre for Biotechnology (IHEC No: IHEC/01/2014/06). All study subjects signed the written informed consent. All methods were performed in accordance with the human ethics guidelines. All subjects had a normal fasting glucose level of 70–110 mg/dL. Their lipid levels were also in the normal range (<200). None of the subjects were on medication nor had any known disease. Briefly, peripheral blood mono nuclear cells (PBMCs) were isolated using Histopaque (Gibco) and incubated with anti CD14 mAb coated microbeads (Miltenyi Biotech). The CD14+ cells which were magnetically retained on the beads were eluted and checked for efficiency using flow cytometry (data not shown). Each experiment was done in triplicates as three independent experiments.

### In vitro monocyte transmigration model

We created an invitro cellular model of monocyte migration, chemotaxis and transmigration using THP cells and immortalized endothelial cell line EA.hy926 as monolayers.

### Monocyte migration assay

Cell migration was evaluated using Transwell inserts (6.5 mm diameter) (Corning Costar) in 24 well plates. Pre-treated THP cells (50,000 cells/well) were added to the upper chamber of the insert. The lower chamber contained 1 mL of normal glucose (NG) or high glucose (HG) (20 mM) RPMI/1% human albumin with or without cyclophilin A as chemokine. LPS (10 µg/mL) was used as positive control throughout the experiments. The plates were incubated for 4–24 h and stained with Giemsa’s stain (Merck Chemicals). The number of cells appearing on the lower face of the filter was recorded in three random fields for each well. The chemotactic index (CI) was calculated as the number of cells migrating towards the test sample divided by the number of cells migrating towards the control medium using light microscopy. The number of cells migrating to the bottom chamber was counted by flow cytometry. The inserts were fixed with 4% para-formaldehyde (PFA) and stained with giemsa’s stain. The cells migrating to the reverse surface of the membrane were counted by light microscopy at 20× magnification in four different random fields. After migration the cells in lower chamber were counted and analyzed directly by flow cytometry (Becton–Dickinson).

### Transendothelial migration

EA.hy926 (4 × 10^5^) cells were cultured on 24 mm diameter polycarbonate membrane (8.0 micron pore size), (Corning Costar). The LPS activated THP-1 cells were seeded in the upper chamber. Cyclophilin A (100 ng/mL) was added in the lower chamber [11]. After incubation, the cells were collected from the lower chamber and analysed by flow cytometry.

### Monocyte to macrophage differentiation

Monocyte to macrophage differentiation was induced with PMA at a final concentration of 50 μg/mL in THP 1 cells cultured in both normal glucose and high glucose in the presence and absence of cyclophilin A for 96 h. Differentiated cells were observed in white field using transmitted detector (TD) of a confocal microscope (Leica SP2 Laser scanning Spectral Confocal system).

### Lipid accumulation by macrophages

THP1 cells (1 × 10^5^) were seeded on rounded cover slips and treated with PMA (50 ng/mL) in 10% high glucose (20 mM glucose) RPMI 1640 medium with or without cyclophilin A. After differentiation, the cells were starved for 24 h and incubated with OxLDL (50 µg/mL) for 24 h [12]. Lipid uptake by macrophages was quantified by oil red O staining, immunocytochemistry and flow cytometry.

### Oil red O (ORO) staining

Cells were fixed with 4% paraformaldehyde (PFA) and stained with oil red O (ORO; Sigma). ORO staining was analyzed by light microscopy (Nikon ECLIPSE *Ti*-*U*, USA) and quantified using Nikon’s comprehensive imaging software NIS Elements. LDL uptake was expressed as the number of ORO-positive cells per 10 × field (average of 3 different areas, n = 3 experiments per group) [12].

### Immunofluoresence assay

Uptake of DiI-oxLDL was studied either with confocal microscopy or fluorescence-activated cell sorting (FACS). Differentiated THP cells were treated with DiI-oxLDL in presence and absence of cyclophilin A for 4 h. Cells were treated with 10 µg/mL of Hoechst 33258 (Sigma) for 5 min. The cells were then placed for confocal microscopy and quantified using microscope imaging software NIS-Elements Viewer.

### Flow cytometry analysis

THP1 differentiated macrophages were incubated with DiI-oxLDL (10 µg/mL) for 24 h with or without cyclophilin A. The specificity of uptake was tested by pretreating the cells with 50-fold excess of unlabeled oxLDL for 30 min and then incubation with dil labeled oxidized LDL for 4 h as described earlier [12]. The intensity was analyzed by fluorescence-activated cell sorting (FACS) (Becton–Dickinson) using FACS Diva v8.0 software. Cells were quantitated by subtracting the cell autofluorescence of the treated samples and expressed as mean fluorescence intensity.

### Immunoblotting analysis

After treatment with OxLDL and cyclophilin A, protein lysates were prepared and separated on SDS-PAGE and resolved proteins were transferred on to nitrocellulose membrane. The membrane was incubated with 1:1000 dilutions of each of the following primary antibodies at 4 °C for 18 h: Mouse anti-cyclophilin A (ab-58144), Mouse anti β Actin (sc-47778), rabbit anti-CD 36 (#14347), Rabbit anti-LOX-1 (ab174316) as per manufacturer’s instructions. The membrane was then incubated with specific secondary antibodies: anti mouse IgG HRP (ab-6789) and anti rabbit IgG-HRP (ab-97051) at a dilution of 1:5000. The proteins were visualized with clarity western ECL substrate. The bands were analyzed by Quantity One 1D image analysis software (Bio-Rad, USA).

### Enzyme linked assay for measurement of cyclophilin A, TNF-α and MCP-1 levels in conditioned medium

Levels of the cytokines TNF-α and MCP-1 as well as cyclophilin A in conditioned medium after treatment was determined with a sandwich immunoassay kit (R&D systems, Uscn Life Science Inc) as per manufacturer’s instructions. The linearity of the kit was assayed by testing samples spiked with a known concentration of proteins and their serial dilutions. All samples were analyzed in duplicate. To maintain assay precision, samples with a CV >12% were excluded.

### Invitro silencing of cyclophilin A gene

THP1 cells (2–4 × 10^5^) were transfected with mission siRNA (7 pmol) using, mission siRNA Transfection Reagent (Sigma Aldrich) for 48 h and TMN 355 for 6 h at 37 °C. Primers used for cyclophilin A mRNA target sequence were 5′-TGGTGTTTGGCAAAGTGAAAGAAGGCATGAATATTGTGGAGGCCATGGAGCGCTTTG -3′. The efficiency of silencing was measured by quantitative real time PCR by measuring relative mRNA expression using ABI Prism 7900HT sequence detection system. Total RNA was isolated from the cells using TRIzol reagent (Sigma), according to the manufacturer’s instructions. RNA yield and purity were calculated by spectrophotometric analysis. Total RNA (10 μg) from each sample was reverse transcribed using random hexamers, dNTPs, and M-MLV reverse transcriptase (Promega). PCR of cDNA was performed using specific primers of cyclophilin A. The reactions were performed in triplicate in 96-well plates at 48 °C, 30 min; 95 °C, 10 min; then 95 °C, 15 s; and 60 °C, 1 min; for 40 cycles. A dissociation curve was constructed by increasing the temperature from 60 to 95 °C at a ramp rate of 2%. A single peak was observed in the dissociation curve of cyclophilin A gene, supporting the specificity of the RT-PCR product. Ct values were used to calculate the expression levels of cyclophilin A gene normalized to endogenous cellular beta 2 microglobulin. The levels of beta 2 microglobulin mRNA was measured in parallel using specific primers from Sigma. The primer sequences of cyclophilin A and beta 2 microglobulin are given in the Additional file 1.

### Treatment of cyclophilin A with its chemical inhibitor, TMN 355

THP 1 differentiated macrophages were treated with TMN 355 at a concentration of 1 µM/mL for 6 h. Time and concentration of TMN 355 treatment was standardized by calculating LC 50 values using MTT assay [13].

### Oxidative stress analysis

PMA differentiated macrophages were incubated with oxidised LDL (50 µg/mL) for 24 h.

### ROS levels by cell ROX Deep Red Reagent using flow cytometry

ROS levels were measured using cell ROX Deep Red Reagent (Thermofisher scientific). THP1 cells were incubated with oxLDL and cyclophilin A to induce oxidative stress. After incubation, cells were treated with cell rox reagent at a final concentration of 5 µM and analyzed by flow cytometry.

### Lipid peroxidation by TBARS assay

Lipid peroxidation by TBARS assay was measured as described earlier [14]. Briefly, THP 1 cells were seeded on six-well plates in a medium containing cyclophilin A and oxidized lipoprotein to induce oxidative stress. After incubation, the cells were washed and resuspended in PBS containing 150 mM butylated hydroxytoluene. The cell suspension was mixed with a solution containing 30% trichloroacetic acid, 0.75% thiobarbituric acid, and 0.5 N hydrochloric acid. Samples were heated at 60 °C for 15 min before they were centrifuged for 8 min at 13,600*g*. The absorbance of the supernatants was measured at 532 nm. TBARS was calculated as malondialdehyde equivalents present using an extinction coefficient of 1.56 × 10^5^ M^−1^ cm^−1.^

### Catalase assay

Catalase activity was measured by catalase assay kit (Abcam,UK) as per manufacturer’s instructions. Briefly, PMA differentiated macrophages were treated with OxLDL in the presence and absence of cyclophilin A (100 ng/mL) for 24 h. Cells were harvested after treatment, washed with cold PBS twice and homogenized with 200 µL of cold assay buffer. To start the reaction 12 µL of 1 mM H_2_O_2_ was added and incubated for 30 min at 25 °C. The unconverted H_2_O_2_ was measured calorimetrically using OxiRed probe at 530 nm using microplate reader (Biorad laboratories, Germany). Catalase activity was calculated as the amount of H_2_O_2_ decomposed per minute at pH 4.5 at 25 °C.

### Statistical analysis

All assays were conducted as three separate experiments in triplicates. Results were recorded as percentage. The differences between various cell treatments were analyzed using one way linear analysis of variance (ANOVA) using GraphPad Prism, by GraphPad Software, Inc. p < 0.05 were considered statistically relevant.

## Results

### Cyclophilin A increases monocyte adhesion and transmigration into the endothelium under high glucose conditions

**Fig. 1:**
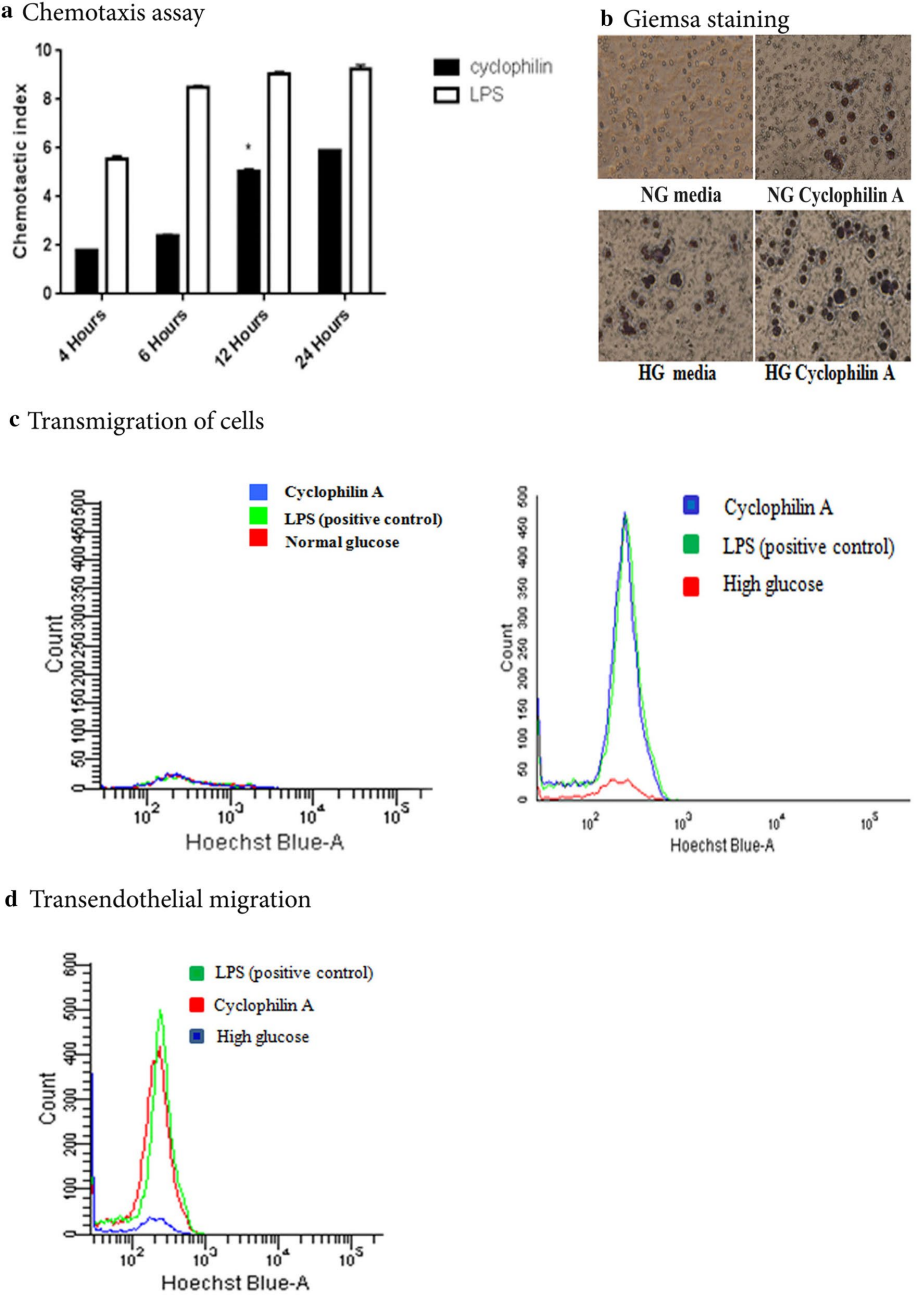
**a** Chemotactic response was analyzed using transwell assays as described in the method section. THP-1 cells were treated with or without cyclophilin A (100 ng/mL) for 4, 6, 12, 24 h. LPS (10 µg/mL) was taken as positive control. **b** Monocytes were cultured on the upper chamber of transwells in normal glucose (NG) and high glucose (HG) in the presence and absence of cyclophilin A (*lower chamber*) for 24 h. The adhered monocytes were stained using Giemsa stain. HG indicates RPMI 1640 culture media primed with high glucose (20 mM/L). (**c**) Flow cytometry analysis of cells after transmigration assays. Transwell experiments were performed using 5.0 micron pore membrane. To test the migration rate, THP 1 cells were seeded on *top* of the transwell and 100 ng/mL cyclophilin A was added to the *bottom* of chambers along with NG (*left panel*)/HG (*right panel*) media. Migration rate of THP cells cultured in HG treated with cyclophilin A were similar to cells treated with LPS alone indicating a strong chemokinetic activity by cyclophilin A. **d** Transendothelial migration of THP 1 cells across monolayer of endothelial cells (EaHy). THP-1 cells were seeded on top of the transwell and 100 ng/mL cyclophilin A was added to the bottom of the chamber. After 24 h of incubation transmigrated cells were counted by flow cytometry using Hoechst (10 µg/mL) as nuclear stain. Transendothelial migration of cyclophilin A treated cells were almost equal to that of positive control, LPS. Data are presented as mean ± SD (n = 3). Chemotactic response was analyzed using two-way ANOVA. p < 0.005 was considered significant

The inflammation cascade begins with adhesion of monocytes to the endothelial cells and transmigration into the intima. We speculated that cyclophilin A under hyperglycemic conditions functions as a chemoattractant and influences the adhesion process. To understand whether cyclophilin A has the potential to increase monocyte adhesion we conducted chemotaxis and transmigration experiments. A dosage of 100 ng/mL cyclophilin A was used for all experiments [10]. Migration was assessed from 4 to 24 h by calculating chemotactic index (Fig. 1a). At an optimum time of 12 h the filters were fixed and stained with Giemsa. The pictures of filter stained with Giemsa reveals increase in number of cells that have adhered to the filter on treatment with high glucose and cyclophilin A (Fig. 1b). Treatment of monocytes cells cultured in high glucose with 100 ng/mL cyclophilin A in the lower chamber markedly increased migration of monocytes compared to untreated cells (Fig. 1c). Transendothelial migration of activated monocytes across monolayer of endothelial cells increased substantially with the addition of cyclophilin A in the lower chamber in high glucose conditions compared to untreated control group. The migrated cells across EaHy cells in normal glucose conditions were very few in number to be counted using flow cytometry. LPS, a known chemokine, was used as positive control (Fig. 1d).

### Cyclophilin A affects monocyte to macrophage differentiation

**Fig. 2:**
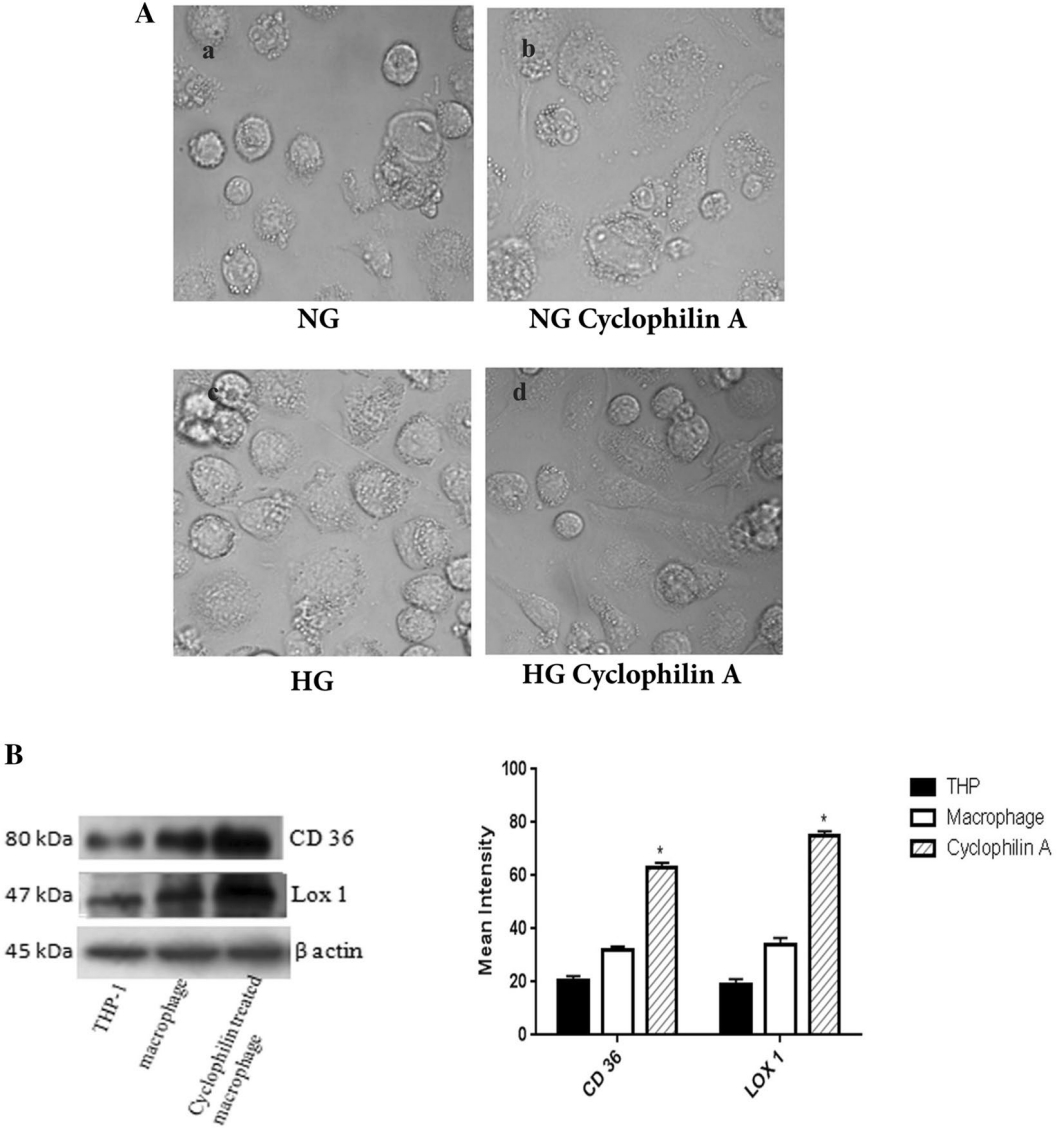
**A** THP cells were cultured for 96 h in 10% RPMI 1640 containing PMA in the presence of cyclophilin A (100 ng/mL) in NG/HG conditions to induce differentiation. The differentiation of monocyte to macrophage was analysed by transmitted detector of confocal microscopy. THP-1 cells incubated in the presence of cyclophilin A and HG exhibited cell flattening and development of cytoplasmic processes. **B** Western blot analysis of scavenger receptor markers, CD 36 and LOX 1 of glucose activated monocyte differentiated macrophages after treatment with cyclophilin A (100 ng/mL). Cyclophilin A treated macrophages in high glucose conditions resulted in a significant increase in LOX-1 and CD36 protein expression. Protein densities of immunoreactive bands measured by the Quantity One 1D analysis software program. Data are presented as mean ± SD (n = 3) and *asterisk* represents p < 0.05

To investigate the effect of cyclophilin A on the process of monocyte differentiation into macrophages, THP cells were cultured for 96 h in 10% RPMI 1640 containing phorbol myristate acetate (PMA) in the presence and absence of cyclophilin A in both normal and high glucose conditions. THP-1 cells incubated in the presence of cyclophilin A were flattened and had developed cytoplasmic processes (Fig. 2A). The expression of scavenger receptor markers, cluster of differentiation 36 (CD36) and lectin-type oxidized LDL receptor 1 (LOX-1) were significantly increased on treatment of monocytes with cyclophilin A (Fig. 2B).

### Cyclophilin A induces oxLDL uptake and stimulates monocyte derived macrophage foam cell formation in vitro

**Fig. 3:**
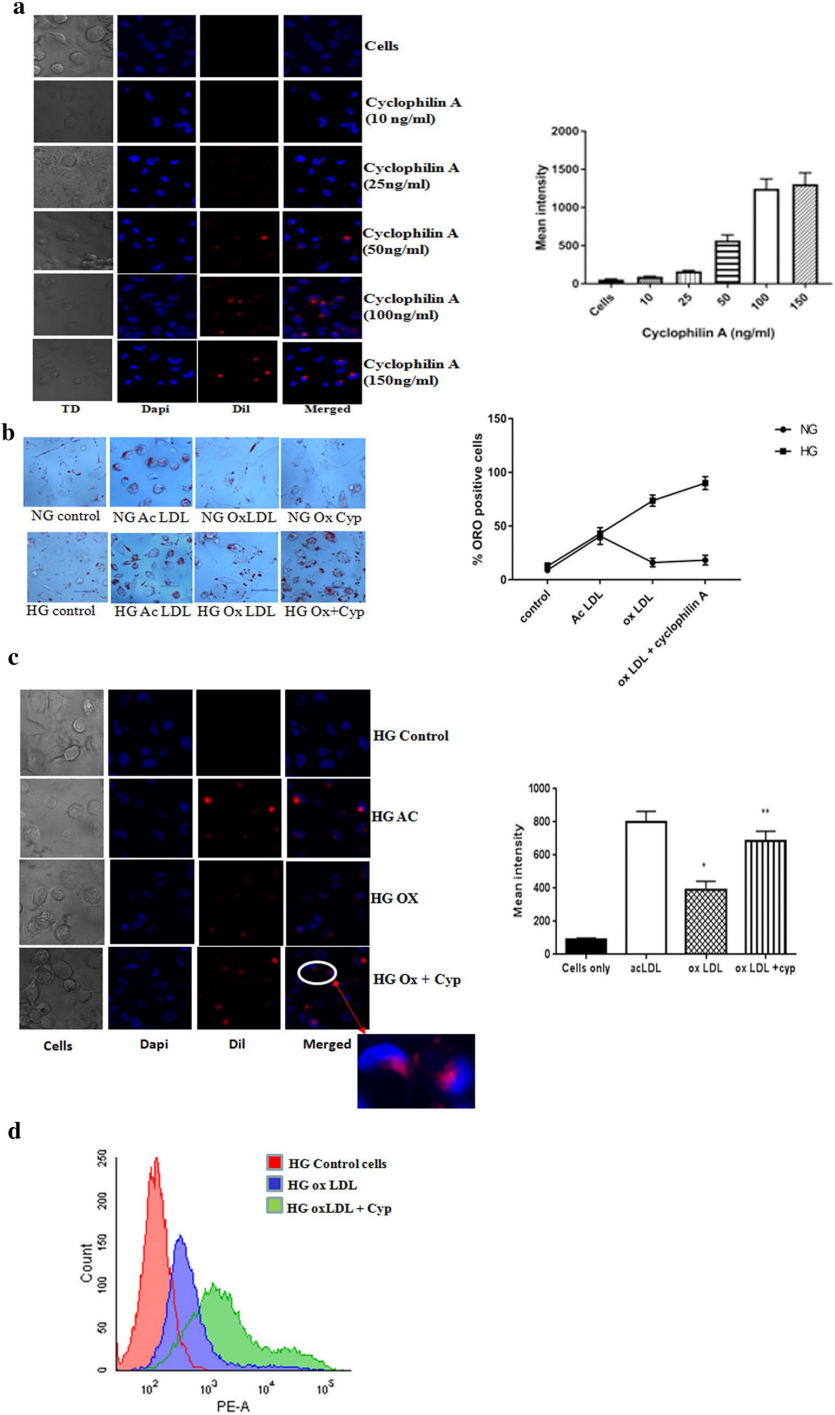
**a** THP cells were treated with cyclophilin A at doses of 10, 25, 50, 100 and 150 ng/mL in the presence of high glucose (HG). HG indicates RPMI culture media primed with glucose (20 mM/L). Lipid uptake was measured using confocal microscopy after treatment with oxidized LDL for 4 h. Maximal effect was observed at a dosage of 100 ng/mL of cyclophilin A. **b** Photomicrographs of lipid laden macrophages stained with oil red O (ORO). THP cells were treated with/without cyclophilin A (100 ng/mL) and oxidized LDL in both normal glucose (NG) and high glucose (HG) conditions for 24 h before staining with ORO. Abundant ORO positivity was seen in cells treated with oxLDL and cyclophilin A cultured in HG conditions. **c** Confocal images of Dil-oxLDL uptake in THP cells differentiated to macrophages in the presence of cyclophilin A (100 ng/mL). Dil-oxLDL uptake is shown in *red*. Cells were counterstained with Dapi (*blue*). *Inset* is the enlarged image of a foam cell showing red coloured lipid droplets. Acetylated LDL (Ac LDL) was taken as the positive control. Mean intensity was quantified using microscope imaging software NIS-Elements Viewer. Cells treated with ox LDL had extensive lipid uptake compared to control cells. **d** Flow cytometric analysis of Dil-OxLDL uptake by macrophages before and after treatment with cyclophilin A in high glucose conditions. Cells were treated with and without cyclophilin (100 ng/mL) for 24 h and then labeled with DiI Ox-LDL for 4 h. The fluorescence intensity was analyzed by FACS using FACS Diva v8.0 software. Cells were quantitated by subtracting the cell autofluorescence of the treated samples and expressed as mean fluorescence intensity

We investigated whether priming of macrophages with cyclophilin A promotes lipid uptake in THP-1 derived macrophages. Lipid endocytosis in macrophages was measured using DiI labeled oxidized low density lipoprotein (DiI OxLDL) treatment for 4 h. Cyclophilin A dose dependently increased the accumulation of lipid droplets in macrophages. From a dose of 50 ng/mL of cyclophilin A onwards, lipid uptake was significantly increased. Maximal effects were observed at a dose of 100 ng/mL. At higher doses no such significant difference was observed (Fig. 3a). This dosage was considered for all further experiments [10]. Cyclophilin A treated macrophages had increased uptake of DiI labeled OxLDL compared with untreated cells, as evident from oil red O (ORO) staining visualized by light microscopy (Fig. 3b), fluorescence microscopy (Fig. 3c) and flow cytometry (Fig. 3d). Cells have an inherent lipid content which stains red on treatment with DiI even in the absence of OxLDL. Exposure to cyclophilin A in the presence of OxLDL markedly increased the uptake of lipids by cells.

### Invitro silencing of Cyclophilin A gene reduces lipid uptake by high glucose primed macrophages

**Fig. 4:**
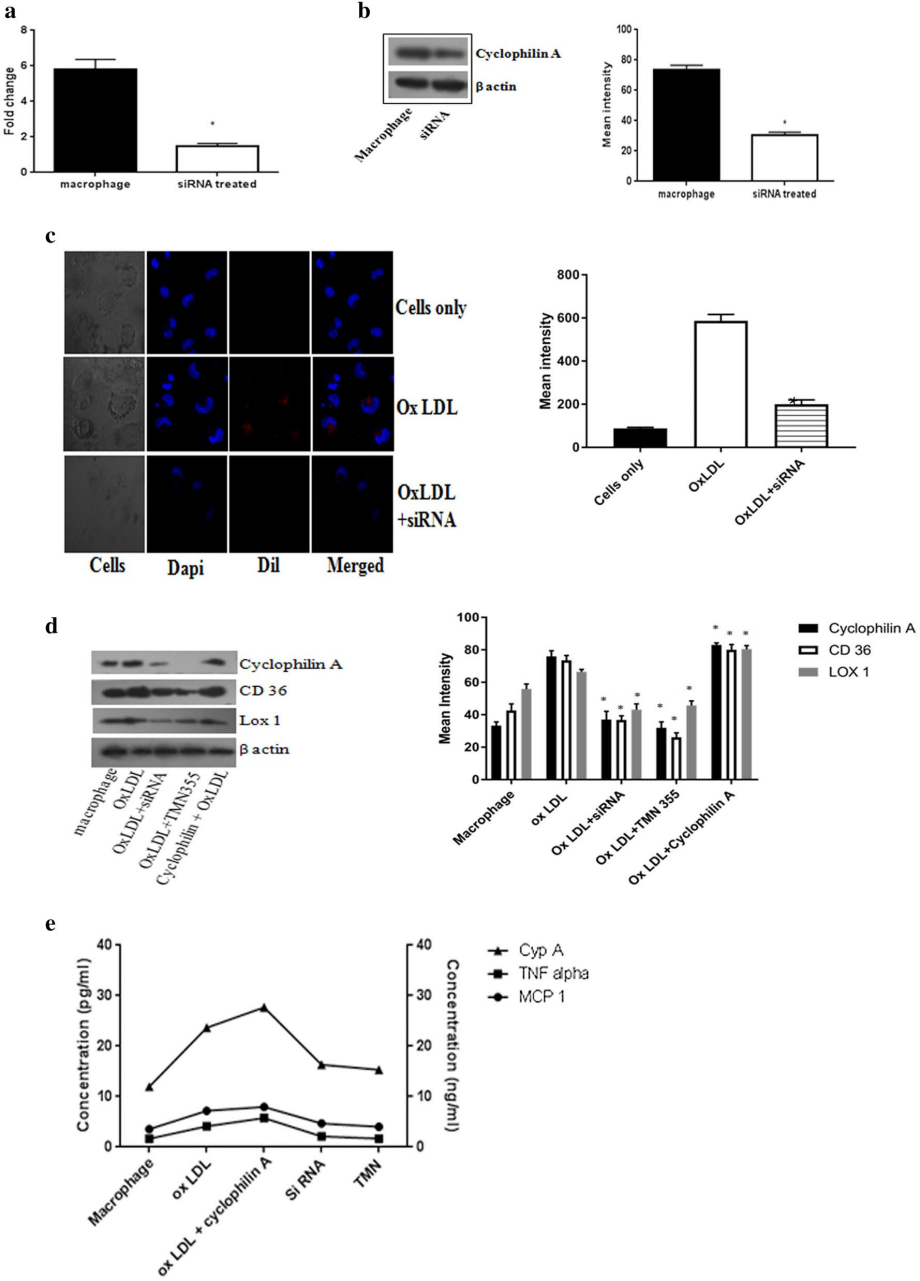
**a** Transfection efficiency was analyzed using real time PCR after treatment of THP differentiated macrophages with mission siRNA (7 pmol) for 48 h. **b** Immunoblotting image of cyclophilin A protein expression after treatment with mission siRNA to confirm transfection. β-actin was used as endogenous control. Protein densities of immunoreactive bands measured by the Quantity One 1D analysis software program. Data are presented as mean ± SD (n = 3) and *asterisk* represents p < 0.05). **c** Confocal images of foam cell formation after transient transfection of cyclophilin A using mission siRNA for 48 h. The transfected cells were treated with DiI conjugated oxLDL. Dapi was used as the nuclear stain. Mean intensity was quantified using microscope imaging software NIS-Elements Viewer, *p < 0.05 was considered significant. **d** Western blot analysis of cyclophilin A after treatment with mission siRNA. Expression of scavenger receptor markers CD36 and LOX-1 were also reduced after siRNA transfection of cyclophilin A. Protein densities of immunoreactive bands measured by the Quantity One 1D analysis software program. Data are presented as mean ± SD (n = 3) and *asterisk* represents p < 0.05. **e** ELISA analysis of conditioned medium after treatment with siRNA and chemical inhibitor of cyclophilin A, TMN 355. Levels of TNF-α (pg/mL), MCP 1 (pg/mL) and cyclophilin A (ng/mL)

To study the effect of intracellular cyclophilin A in lipid uptake by monocytes and extracellular secretion of cyclophilin A, we attempted to silence the cyclophilin A gene in macrophages. We used mission small interfering RNA (siRNA) to knockdown cyclophilin A in monocyte differentiated macrophages using MISSION siRNA transfection reagent. There was 70–85% transfection efficiency, as evident from quantitative analysis by real time PCR (Fig. 4a). The gene silencing effect of cyclophilin A mission siRNA was confirmed at the protein level by western blotting (Fig. 4b). Knockdown of cyclophilin A with siRNA resulted in significant reduction (p < 0.05) in lipid uptake by macrophages (Fig. 4c). We also confirmed that cyclophilin A could be suppressed by siRNA within 48 h and decrease the expression of scavenger receptor markers, CD 36 and LOX 1 eventually reducing lipid uptake by macrophages (Fig. 4d). Expression of CD 36 and LOX 1 remained increased in macrophages which were treated with cyclophilin A.

Parallely with reduction of cyclophilin A protein expression, the levels of other inflammatory cytokines such as monocyte chemoattractant protein-1 (MCP 1), tumor necrosis factor alpha (TNF α) as well as extracellular cyclophilin A determined by enzyme-linked immunosorbent assay (ELISA) were reduced in cyclophilin A silenced monocyte differentiated macrophages (Fig. 4e). This is in contrast to the increased levels of cytokines in non-silenced cells treated with OxLDL and cyclophilin A (Fig. 4e).

### TMN355, a chemical inhibitor of cyclophilin A reduces foam cell formation and cytokine secretion

**Fig. 5:**
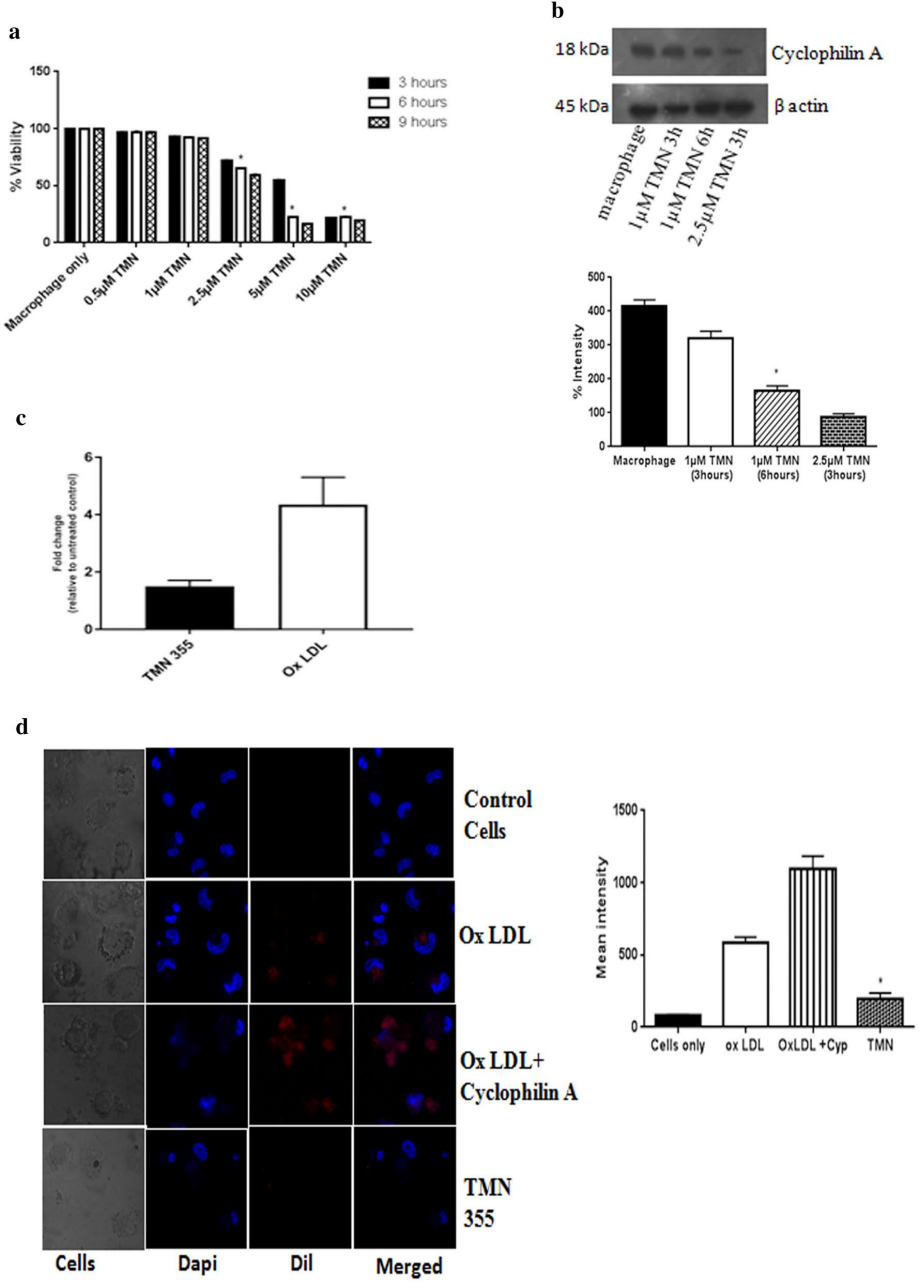
**a** Effect of TMN355 on cell viability was measured using MTT assay in a time and dose dependent manner. TMN 355 concentrations of 0.5, 1, 2.5, 5 and 10 μM were tested at 3, 6 and 9 h. **b** Effect of TMN 355 on protein level expression of cyclophilin A. Protein densities of immunoreactive bands measured by the Quantity One 1D analysis software program. Data are presented as mean ± SD (n = 3) and *asterisk* represents p < 0.05. **c** mRNA level of cyclophilin A in the presence and absence of TMN 355 after treatment with oxidized LDL. **d** Confocal microscopy images of Dil OXLDL uptake in THP differentiated macrophages after treatment with TMN 355 for 6 h. Mean intensity was quantified using microscope imaging software NIS-Elements Viewer, *p < 0.05 was considered significant

Here, we tested the effect of a chemical inhibitor of cyclophilin A on lipid uptake by monocyte derived macrophages. TMN 355 (2-Chloro-N-[(9H-fluoren-9-ylamino)carbonyl]-6-fluorobenzamide) is a potent inhibitor of cyclophilin A which contains an amide linker that contributes to inhibitory activity via forming 2–3 hydrogen bonds with residues Arg55, Gln63, and Asn102 around the “saddle” between the two sub-binding pockets of cyclophilin A and thus inhibiting the cis–trans isomerase activity of cyclophilin A [15]. It is 27 times more potent than cyclosporine A and is void of the immunosuppressive function of cyclosporine. Being a chemical compound, we initiated this study with a cell viability assay using MTT (3-(4,5-Dimethylthiazol-2-yl)-2,5-Diphenyltetrazolium Bromide) in a time and dose dependent manner. A dose of 1 μM TMN 355 was found to inhibit cyclophilin A expression without affecting cell viability (Fig. 5a). Hence, 1 μM TMN 355 was used to inhibit cyclophilin A after 6 h of activation.

Interestingly, the treatment with TMN 355 resulted in 75.9% reduction of cyclophilin A protein expression (Fig. 5b). This indicates that TMN 355 affects not only the enzyme activity but also protein level expression. To further understand the mechanism of action of TMN355 on cyclophilin A protein expression, we measured transcript levels of cyclophilin A in TMN 355 treated macrophages using real time quantitative PCR. The mRNA levels of cyclophilin A was decreased in TMN 355 treated macrophages (Fig. 5c) in agreement with the protein expression in Fig. 5b [16].

We next examined the biological effect of TMN 355 on lipid uptake by macrophages. A dose of 1 μM TMN 355 caused a marked decrease in lipid uptake as evident from immunofluoresence assay (Fig. 5c). The ability of TMN 355 to reduce foam cell formation was also determined by immunoblotting experiments (Fig. 4c). Similar to the observations in knockdown experiments using siRNA, the levels of proinflammatory cytokines TNF α, MCP1 and extracellular cyclophilin A were significantly reduced in monocyte derived macrophages on inhibition using TMN 355 (Fig. 4d).

### Cyclophilin A induces foam cell formation in human monocytes

**Fig. 6:**
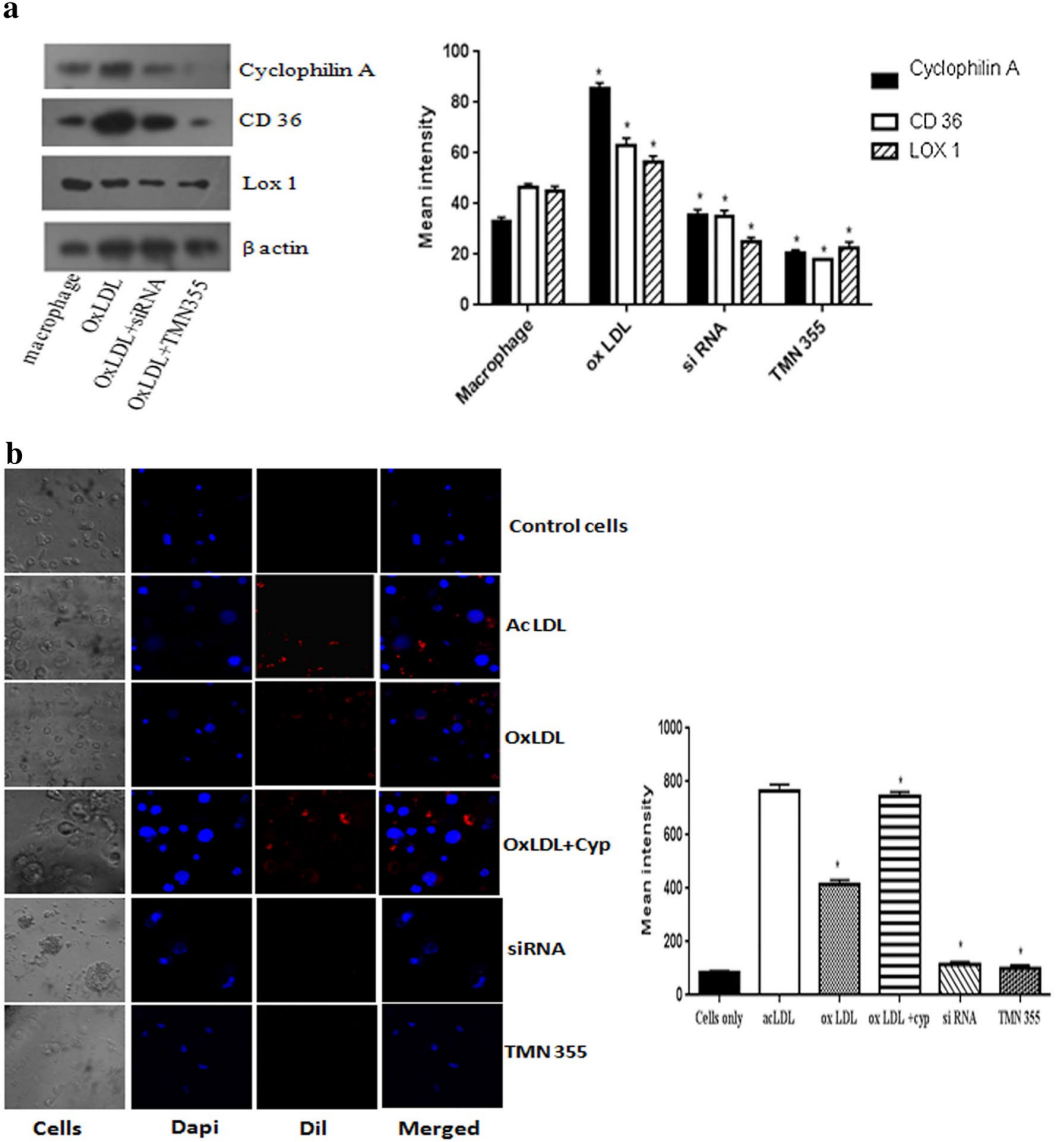
Monocyte to macrophage differentiation and lipid uptake in human monocytes isolated from blood. **a** Representative images of western blot analysis of protein level expression of cyclophilin A, CD 36 and LOX 1 in glucose activated human monocytes during foam cell formation. Protein densities of immunoreactive bands measured by the Quantity One 1D analysis software program. Data are presented as mean ± SD (n = 3) and *asterisk* represents p < 0.05. **b** Confocal images of Dil-oxLDL uptake in human monocyte cells differentiated to macrophages. Macrophages were incubated with DiI-ox LDL and acetylated LDL (10 μg/mL for 4 h) before and after treatment with cyclophilin A inhibitor or siRNA and examined using a 559 nm filter. Dil-oxLDL uptake is shown in* red* and counterstained with Dapi (*blue*). Mean intensity was quantified using microscope imaging software NIS-Elements Viewer, *p < 0.05 was considered significant

The effect of cyclophilin A on monocyte differentiation into macrophages and foam cell formation was further studied on freshly isolated human monocytes cultured for 96 h in RPMI 1640 in the presence and absence of cyclophilin A. Monocytes were differentiated into macrophages and then treated with OxLDL for 4 h. Cyclophilin A treatment resulted in the increased expression of CD 36 and LOX1 in cells primed with OxLDL. There was a marked decrease in the expression of CD36 and LOX1 when the macrophages were treated with siRNA or TMN 355 (Fig. 6a). The uptake of DiI OxLDL, analyzed by confocal microscopy, was increased in cyclophilin A treated monocytes, which was effectively reduced by 65.3% on siRNA treatment and 77% by chemical inhibition using TMN 355 (Fig. 6b), (p < 0.005).

### Cyclophilin A induces oxidative stress during foam cell formation in hyper glycemic conditions

**Fig. 7:**
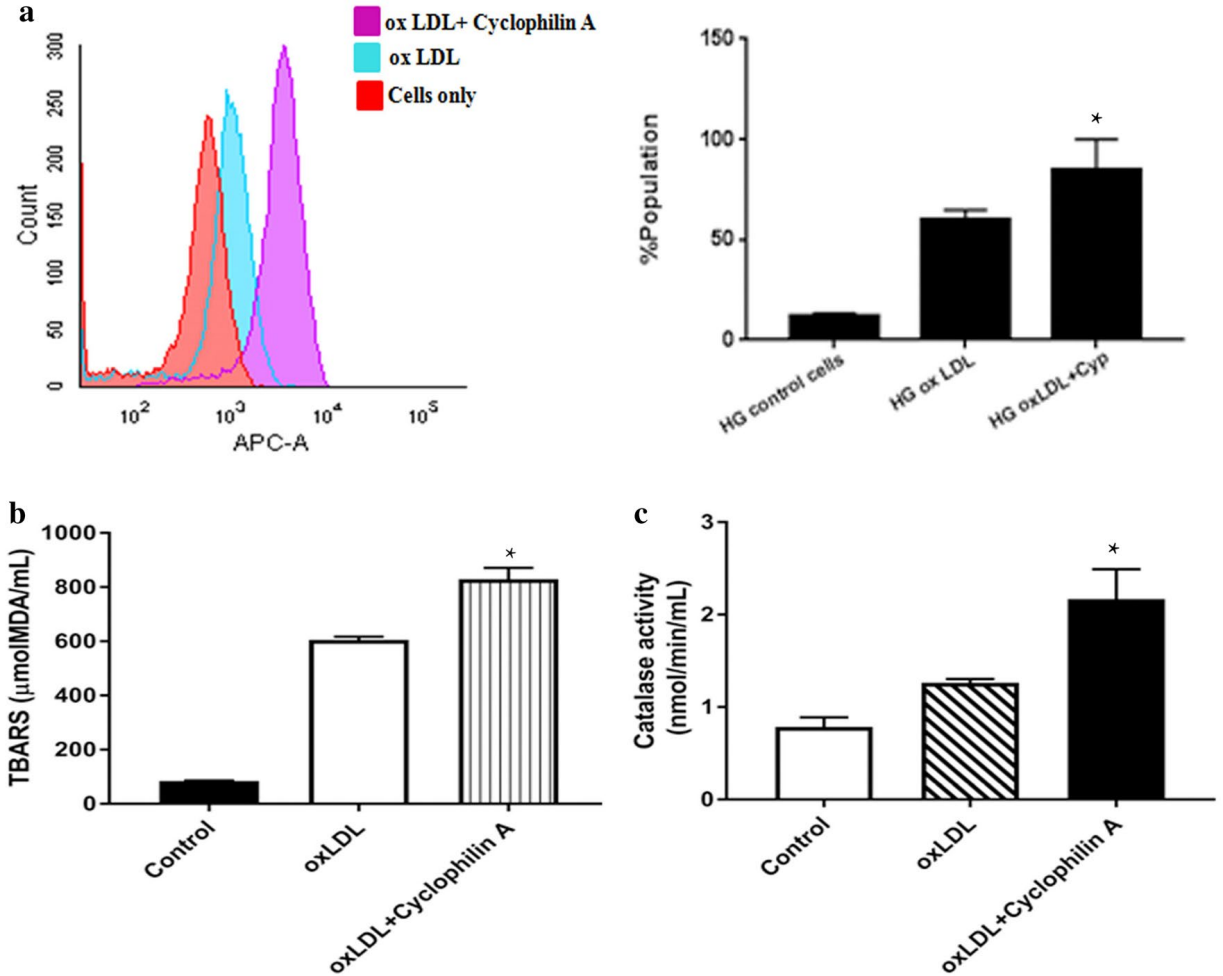
**a** Flow cytometric analysis of Cell Rox (5 µM) uptake by THP differentiated macrophages cultured in high glucose (20 mM/L) during foam cell formation. Monocyte differentiated macrophages were treated with cell ROX reagent at a final concentration of 5 µM. Cells treated with cyclophilin A had increased oxidized LDL induced cytosolic ROS production. The *right panel* shows fluorescence intensity analyzed by FACS using FACS Diva v8.0 software. Cells were quantitated by subtracting the cell autofluorescence of the treated samples and expressed as mean fluorescence intensity. **b** Thiobarbituric acid-reactive substances (TBARS) assay to assess the levels of lipid peroxides in medium of cultured THP1 cells treated with or without cyclophilin A. **c** Catalase enzyme activity assay of cells treated with oxLDL in the presence and absence of cyclophilin A. Data are presented as mean ± SD (n = 3) and *asterisk* represents p < 0.05

We observed an increase in secretion of inflammatory cytokines such as MCP 1, TNF-α as well as cyclophilin A by monocyte differentiated macrophages primed with OxLDL. This led us to investigate whether cyclophilin A activates cells to produce reactive oxygen species (ROS). The cell ROX Deep Red Reagent detects superoxide localized in the cytoplasm in living cells. To investigate the effect of cyclophilin A in lipid peroxidation, we treated the monocyte differentiated macrophages with cell ROX reagent at a final concentration of 5 µM. Lipid peroxidation was assayed by flow cytometry using fluorescent probes cell ROX. As illustrated in Fig. 7a, cyclophilin A substantially increased OxLDL induced cytosolic ROS production (p < 0.0001). We also measured the level of malondialdehyde (MDA), another well studied marker of lipid peroxidation. MDA is reported to impair the interaction between oxidized lipoprotein and macrophages thereby promoting atherosclerosis [17]. It is quantified using a calorimetric assay based on the reaction between MDA and thiobarbituric acid (TBA). We used the TBARS assay to assess oxidative stress on treatment of macrophages with cyclophilin A. As illustrated in Fig. 7b, treatment with cyclophilin A significantly increased the amount of lipid peroxides in the macrophages (p < 0.0001). The production of ROS is regulated by antioxidant enzymes such as catalase [18]. Excessive ROS production which cannot be buffered by antioxidant enzyme results in oxidative stress. Catalase functions to catalyze the decomposition of hydrogen peroxide to water and oxygen. Catalase activity is reversibly proportional to the signal. Our experiment with catalase assay kit (abcam) indicate that oxidative stress increases on treatment of macrophage with oxidized lipoprotein and cyclophilin A (p < 0.0001) (Fig. 7c).

## Discussion

In this report we provide evidences that cyclophilin A in high glucose conditions can function as a chemoattractant increasing monocyte adhesion to the endothelium and enhance transmigration and differentiation of monocytes to foam cells. We also demonstrate that cyclophilin A induces lipid uptake by macrophages and that inhibition of cyclophilin A can prevent foam cell formation through an oxidative stress induced pathway. Taken together, our results suggest that cyclophilin A is an important regulator of vascular inflammation in diabetes mellitus.

Hyperglycemia causes damage to the vascular wall through several mechanisms that include: increased flux of glucose through the polyol pathway; activation of protein kinase C (PKC) isoforms; overactivation of the hexosamine pathway; increased intracellular formation of advanced glycation end products (AGEs) and expression of its receptors and ligands [19]. Endothelial cells cultured in high glucose demonstrate abnormal cell cycles, slow replication and increase in apoptosis [20]. All these pathways lead to increased oxidative stress. ROS decreases the metabolism of glucose through glycolysis, and increases the flux via the alternate pathways which activate damaging mechanisms such as PKC pathway, hexosamine and polyol pathways and AGE formation. These mechanisms eventually increase oxidative stress and vascular permeability [19].

Initiation and progression of early atherosclerosis lesions are different from the mechanism that causes thrombotic plaques [19, 21, 22]. Early atherogenesis involves the activation of endothelial cells; recruitment of monocytes; cholesterol loading of cells to form foam cells; and migration of smooth muscle cells to the intima. In contrast, advanced plaque progression is influenced by processes that promote necrosis and thinning of the fibrous cap over the lesion [23]. Monocyte-derived macrophages play important roles in all phases of atherosclerosis [24]. In early lesions, monocytes are recruited to the intima by activated endothelium. In the intima they become cholesterol-loaded foam cells and participate in proatherogenic processes such as inflammation, secretion of proteases and procoagulant/thrombotic factors, and formation of the necrotic core of clinically dangerous lesions.

When macrophages are exposed to high glucose concentrations invitro, inflammation is induced as evident from experiments in diabetic mice and subjects [25, 26]. A way to reduce inflammation is by blocking cytokine action. Inflammatory intermediaries, such as TNF-α, interleukins, leptin, MCP-1, C-reactive protein (CRP), fibrinogen, angiotensin, retinol binding protein-4, and adiponectin are involved in the action of insulin and maintenance of inflammatory response [27, 28]. These cytokines are involved in insulin resistance and development of atherosclerosis. TNF-α, ROS and free fatty acids activate inflammatory pathways and modulate the expression of numerous genes involved in insulin resistance [29, 30]. Enhanced monocyte activation in diabetes induces activation of various cytokines and chemokines as well as secretion of monocyte proteins, relevant to the pathogenesis of atherosclerosis. One such secreted protein is cyclophilin A [2] which is found both in extracellular and intracellular forms. Earlier studies have shown a strong association between secreted extracellular cyclophilin A and vascular disease [8, 10]. The increased expression of intracellular cyclophilin A has been reported in several stages of atherosclerosis. Deficiency of cyclophilin A has been associated with decreased low density lipoprotein uptake, vascular cell adhesion molecule 1 (VCAM 1) expression and apoptosis in Apolipoprotein deficient mice. Cyclophilin A decreases endothelial nitric oxide expression though Kruppel like factor 2 transcription repression in endothelial cells [31]. Cyclophilin A has been suggested as a secreted oxidative stress induced factor from vascular smooth muscle cells [32, 33]. Secreted cyclophilin A stimulates extracellular signal regulated kinase ½ (ERK ½) and JAK/STAT pathway invitro [5, 34].

In our experiments with THP-1 cells cultured in high glucose conditions (20 mM/L, equivalent to 360 mg/dL of blood sugar levels in humans), we observed an increased adhesion of monocytes to the transwell inserts in the presence of cyclophilin A. Extracellular cyclophilin A is reported to trigger the activation of endothelial cells to produce various adhesion molecules to attract monocytes [35–37]. These adhesion molecules along with other proinflammatory cytokines and extracellular cyclophilin A induce the adhesion of monocytes to the vascular endothelium. As monocyte pass through the endothelial layer and differentiate, they produce various bioactive factors such as TNF-α, interleukins, MCP 1 etc., which promote inflammation in atherosclerosis. Cyclophilin A is associated with this activation process of monocytes [38–40]. We discovered that migration elicited in the presence of cyclophilin A was equal to that elicited when lipopolysaccharides (LPS) was used to induce cell migration suggesting that cyclophilin A exhibits similar chemokine activity as MCP-1 and TNF-α in high glucose conditions. Cyclophilin A induces accumulation of other leukocytes, such as neutrophils, eosinophils, T lymphocytes within atheroma lesions, which facilitate their adhesion to the injured endothelium [41, 42]. Cyclophilin A induces a rapid inflammatory response characterized by neutrophil influx, when injected in vivo [43]. Activated T lymphocytes and monocytes, which are critical for the development of atherosclerosis, show high chemotaxis to extracellular cyclophilin A and its receptor CD147 [37]. Although the exact mechanism is unclear, cyclophilin A is implicated in chemokine receptor C-X-C chemokine receptor (CXCR) 4-mediated chemotaxis by regulating mitogen-activated protein kinase (MAPK) [44]. Cyclophilin A strongly upregulated stromal cell derived factor-1α (SDF-1) and its specific receptor CXCR4 gene expression in C57B1/6J mice and promoting neovascularisation in ischemic limb injury by increasing the recruitment of inflammatory cells into ischemic limbs [45]. Taken together these results implicate cyclophilin A as a major chemokine on par with other inflammatory cytokines modulating atherogenesis in diabetes.

The next step after adhesion is migration of monocytes into the sub endothelial space where they differentiate into macrophages. This step can be reproduced in the cell culture using EaHy.926 as an endothelial mono layer and THP-1 cells as migratory cells. Cyclophilin A added in the lower chamber markedly increased transmigration of monocytes and differentiation into macrophages. High glucose per se enhances transendothelial migration of monocytes, possibly by disruption of endothelial adherens junctions and promoting movement of monocytes through the protein kinase C mediated tyrosine phosphorylation of vascular endothelial cadherin [46, 47]. It is possible that extracellular cyclophilin A expressed by high glucose activated monocytes stimulates the endothelial cells to facilitate the transendothelial migration of monocytes by inducing the expression of adhesion molecules via MAPK signalling pathway [35, 48]. The differentiation of monocyte to macrophages is associated with increased expression of scavenger receptors, LOX-1 and CD 36 [49]. In our study, treatment of THP-1 cells and human monocytes with cyclophilin A resulted in increased expression of CD36 and LOX-1. Subsequently when cyclophilin A was inhibited with siRNA and TMN355, the expression of these scavenger receptors was reduced. The expression of CD36 and LOX-1 are dependent on oxidative stress induced by vascular injury and hyperglycaemia. Further, cyclophilin A being a molecular chaperone may be required for folding and/or transporting LOX-1 and CD 36 to the cell membrane. This could be the reason for reduced the expression of scavenger proteins in the absence of cyclophilin A [50]. Cyclophilin A is also reported as a pro apoptotic cytokine which triggers apoptosis of endothelial cells [38]. Cyclophilin A deficiency is associated with a marked decrease in apoptosis of endothelial cells. Studies using macrophages from patients with type 2 diabetes mellitus and diabetic mice reveal that macrophages in diabetic conditions have an increased activation of pro atherogenic pathways [51, 52]. Mice models which lacks CD 36 have reduced oxLDL internalisation and are less prone to foam cell formation in vitro [53, 54].

Foam cell formation on treatment with OxLDL is well documented [55]. We focussed on the influence of cyclophilin A in OxLDL uptake by monocyte derived macrophages. Cyclophilin A increased the lipid uptake as evident from our qualitative and quantitative assays (p < 0.05). Inhibition of cyclophilin A by silencing or chemical inhibitors strongly reversed this process by reducing lipid uptake. Possibly siRNA mediated knockdown may have removed the target mRNA of cyclophilin A and consequently the protein from the macrophages resulting in decreased lipid uptake. The chemical inhibition by TMN 355 may have blocked the function of the protein thus resulting in a similar effect. These results suggest the importance of cyclophilin A in the process of foam cell formation in macrophages. There are reports that apolipoprotein E deficient mice (apoE) fed with high-cholesterol diet develop severe atherosclerosis compared to apoE and cyclophilin A-deficient mice, indicating that cyclophilin A deficiency in vivo decreases atherosclerotic lesions [56]. We observed that silencing of cyclophilin A also decreased lipid uptake by macrophages in high glucose conditions. Interestingly, the extracellular cyclophilin A during this process mirrored the intracellular cyclophilin A expression. This is in agreement to our earlier report on cyclophilin A expression in circulating monocytes [3] in patients with type 2 diabetes mellitus.

Macrophages have increased expression of inherent cyclophilin A probably because of more redox activity in the sub endothelial space [57, 58]. At the same time, the levels of extracellular cyclophilin A were also higher as evident from immunoassays. Studies on lipid uptake in atherosclerosis models have reported increased expressions of matrix metalloproteinase 9 (MMP9) in cyclophilin A mediated foam cell formation. MMP-9 secretion was reported significantly reduced when treated with the cyclophilin A inhibitor NIM811 [59]. Several experiments have demonstrated that the expression of macrophage colony stimulating factor (M-CSF) and MMPs are effectively retarded after inhibition of cyclophilin A. The M-CSF plays an important role in differentiation of monocytes to lipid laden macrophages by increasing expression of scavenger receptors. These function by internalising modified lipoproteins to form the initial lesion [35, 48]. A reported ligand of cyclophilin A, extracellular matrix metalloproteinase inducer (EMMPRIN) has been found in atherosclerotic plaques of ApoE deficient mice which suggest that cyclophilin A may play a role both in initiation of lesion as well as vulnerability of the plaque for rupture [59].

Macrophages in an attempt to eliminate excessive lipids proliferate and form more foam cells. Cyclophilin A is crucial for macrophage proliferation which is dependent on macrophage M- CSF. This proliferation is dependent on regulation by cyclophilin A [48]. As a consequence of increase in extracellular and intracellular cyclophilin A an increasing number of foam cells accumulate eventually forming a vascular lesion. This entire process is instigated by redox activity. Cyclophilin A has been suggested as an oxidative stress induced factor which mediates the production of ROS in circulation as well as in the intima [31, 58]. Our findings in this study support this view. The oxidative stress levels were increased in macrophages treated with cyclophilin A. Formation of ROS and lipid peroxidation activity may be stimulated by hyperglycemia as well as the presence of increased intracellular and extracellular cyclophilin A. Other factors that could induce ROS are hypoxia, secreted factors such as tyrosine kinase receptors etc. [60]. Intracellular ROS also increases expression and secretion of cyclophilin A in vascular smooth muscle cells (VSMCs) [56]. Our results suggests that increased ROS levels due to intracellular cyclophilin A activity can be detected as early as foam cell formation of monocytes which is the primary step for plaque initiation in the arterial wall. A limitation of our study is the in vitro nature of our experiments, however we have shown increased lipid uptake in the presence of cyclophilin A by human monocytes isolated from peripheral blood mononuclear cells. We have also demonstrated that silencing of cyclophilin A in human monocytes can attenuate lipid uptake to a large extent.

**Fig. 8:**
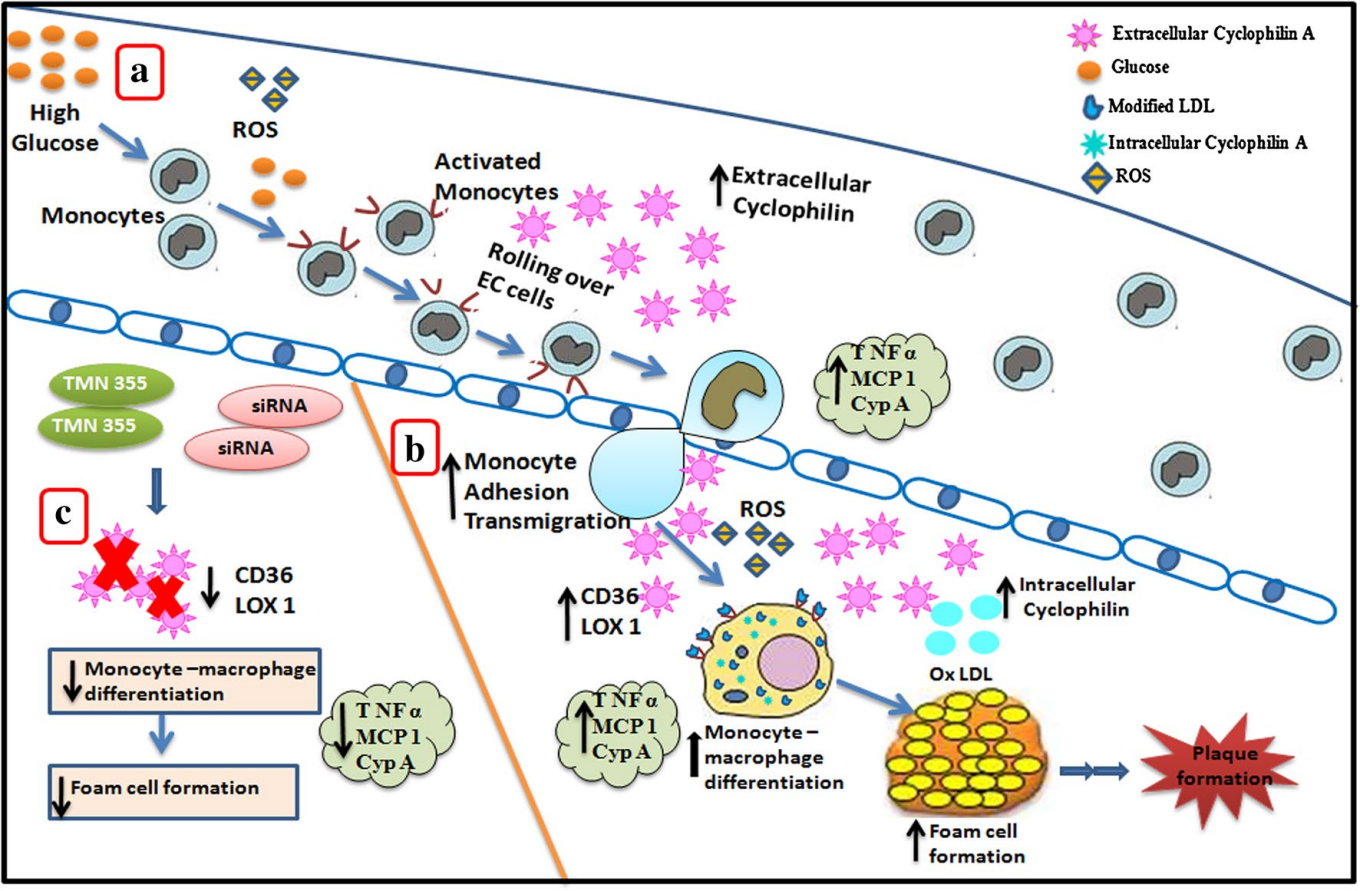
**a** Oxidative modification of lipoprotein in the presence of high glucose in the vascular system activates monocytes as well as the endothelial cells facilitating adhesion of circulating monocytes. Activated endothelial cells secrete chemokines and promote monocyte rolling, tethering and then transmigration into the subendothelial space. **b** These monocyte then firmly adhere to the endothelial cells and differentiate into macrophages. Macrophages engulf oxidised lipoproteins to form foam cells. These modifications incite an inflammatory response and result in an increase in the levels of cytokines such as MCP-1, TNF-α and extracellular cyclophilin A. **c** When monocyte derived macrophages are treated with either siRNA or chemical inhibitor TMN 355, to silence/inhibit cyclophilin A it leads to a decrease in expression of CD36 and LOX-1 indicating reduced monocyte-macrophage differentiation as well as reduced lipid uptake. The levels of inflammatory cytokines, MCP-1, TNF-α and cyclophilin A also decreases. This highlights the role of cyclophilin A in promoting vascular inflammation in hyperglycemia

Thus, cyclophilin A in both extracellular and intracellular forms promotes atherogenesis in hyperglycaemic conditions. Our results implicate cyclophilin A at various stages of atherogenesis such as monocyte adhesion, transmigration, monocyte to macrophage differentiation and foam cell formation (Fig. 8). Possibly, high glucose levels in circulation increases redox activity and facilitates adhesion of circulating monocytes to the endothelium and its subsequent differentiation into macrophages in the sub endothelial space. The presence of extracellular cyclophilin A secreted from glucose activated monocytes in circulation further enhances this adhesion and transmigration process. Increased intracellular cyclophilin A within macrophages increases scavenger receptors such as CD 36 on its surface owing to the chaperone activity of cyclophilin A. This accelerates accumulation of modified lipoproteins in macrophages subsequently forming foam cells. This may trigger an inflammatory response increasing cytokine levels leading to secretion of the intracellular cyclophilin A into the circulation in its extracellular form. However the exact mechanism of this secretory process requires further contemplation.

In our attempt to understand the mechanism underlying the process of cyclophilin A induced foam cell formation by monocytes activated by high glucose we found that cyclophilin A upregulates scavenger receptors and increases redox activity as well as levels of proinflammatory cytokines leading to increase in lipid uptake by macrophages. This is the first study which evaluates cyclophilin A as a regulatory protein in vascular lesion formation in high glucose conditions.

To summarize, we describe here three mechanisms by which cyclophilin A promotes atherogenesis in diabetes mellitus. First, cyclophilin A acts as a chemokine and increases adhesion and transmigration of glucose activated monocytes into the sub endothelial space. Second, cyclophilin A modulates monocyte to macrophage differentiation by regulating the expression of scavenger receptors. Third, inhibition of cyclophilin A reduces oxLDL uptake which is mediated through oxidative stress. All these mechanisms together contribute to vascular inflammation in hyperglycemia. In conclusion, our results demonstrate that high concentrations of glucose results in increased monocyte adhesion, transmigration, differentiation and formation of foam cells through a cyclophilin A mediated inflammatory process. It is possible that targeting cyclophilin A activity using inhibitory agents or molecules may retard progression of atherosclerotic lesions in diabetes. In this context, cyclophilin A gains clinical significance as a target for treatment strategy in preventing vascular complications in patients with diabetes.

## Additional file

**Additional file 1.** Primer sequences of cyclophilin A and beta-2-microglobulin for real time PCR.

## Abbreviations

Dil OxLDL: dil labeled oxidized low density lipoprotein
TNF α: tumor necrosis factor alpha
MCP 1: monocyte chemoattractant protein-1
CD 36: cluster of differentiation 36
LOX 1: lectin-type oxidized LDL receptor 1
TMN 355: 2-Chloro-N-[(9H-fluoren-9-ylamino)carbonyl]-6-fluorobenzamide
siRNA: small interfering RNA
ELISA: enzyme-linked immunosorbent assay
MTT: (3-(4,5-Dimethylthiazol-2-yl)-2,5-Diphenyltetrazolium Bromide)
PMA: phorbol myristate acetate
CRP: C reactive protein
VCAM 1: vascular cell adhesion molecule 1
ERK: extracellular signal-regulated kinases
MMP 9: matrix metalloproteinase 9
HEPES: (4-(2-hydroxyethyl)-1-piperazineethanesulfonic acid
LPS: lipopolysaccharides
PFA: paraformaldehyde
PBS: phosphate-buffered saline
BSA: bovine serum albumin
TBST: Tris-Buffered Saline and Tween 20
ORO: oil red O staining
dNTPs: nucleotide triphosphates
TBARS: 2-Thiobarbituric Acid Reactive Substances
MDA: malondialdehyde
TBA: thiobarbituric acid
