# Supplementary material for: Cyclophilin A enhances macrophage differentiation and lipid uptake in high glucose conditions: a cellular mechanism for accelerated macro vascular disease in diabetes mellitus
Source: Cardiovasc Diabetol. 2016 Nov 3;15:152. doi: 10.1186/s12933-016-0467-5 (PMC5094075; doi:10.1186/s12933-016-0467-5)
Supplement: Supplementary file 1 — Additional file 1. Primer sequences of cyclophilin A and beta-2-microglobulin for real time PCR. [file 12933_2016_467_MOESM1_ESM.doc]

**SUPPLEMENTAL MATERIAL**

Primer sequences of cyclophilin A and beta-2-microglobulin for real time PCR

| Cyclophilin A | |
| --- | --- |
| Forward primer | 5’-CACCGCCGAGGAAAACCGTGT-3’ |
| Reverse primer | 5’-GGGACCTTGTCTGCAAACAGCTCA-3’ |

| beta-2-microglobulin | |
| --- | --- |
| Forward primer | 5’ - CCAGCGTACTCCAAAGATTCAG - 3’ |
| Reverse primer | 5’ - GTAAGTCAACTTCAATGTCGGATG - 3’ |
